# Monitoring rowers to determine under-performance

**DOI:** 10.1186/2052-1847-7-S1-O15

**Published:** 2015-08-11

**Authors:** Jürgen Michael Steinacker, Martina Zügel, Uwe Schumann, Katja Machus, Marion Schneider, Hohmann Harald, Mahdi Sareban, Gunnar Treff

**Affiliations:** 1 Division of Experimental Anesthesiology; University of Ulm, Ulm, Germany; 2 Division of Sports- und Rehabilitation Medicine, University of Ulm, Ulm, Germany

## Introduction

Over the past few years, training load and performance in competition has continuously increased and is fraught with risk to result in the accumulation of fatiguing conditions. The coach determines training success by controlling boat speed, performance, power and soft parameters like stability of rowing technique, capacity to teamwork, and mood state. However, in team sports like rowing, monitoring of individual rowers fatigue to optimize regeneration is difficult. While training should voluntarily cause acute fatigue, the accumulation of training leads to more severe fatigue, which is called “*overreaching”*. When fatigue is more prolonged and recovery is impaired, the condition is defined as *“non-functional overreaching”* ending in a primarily unexplained, long-term, and unplanned decreasing performance, a condition defined as *“Unexplained Underperformance Syndrome” (UUPS*) or *“overtraining syndrome”* (OTS). Good standards and appropriate markers for diagnosis and treatment are currently lacking.

## Clinical signs of UUPS / OTS

Athletes present with the key symptoms of prolonged underperformance and/or reduced trainability or disturbed regeneration following a period of heavy training load. Mood disturbances like fatigue, lethargy, exhaustion, sleep disturbances, and increased susceptibility to infections are present; athletes experience increased levels of perceived stress, decreased levels of regeneration and burnout. Often, athletes report about previous upper-respiratory-tract infections. Physical signs include muscle pain, non-specific irritation of the mucous membranes, increased heart rate at rest and during a given workload, performance, and maximum oxygen uptake and maximum lactate levels are decreased.

## Pathogenesis

The syndrome has been linked to carbohydrate metabolism, decreased levels of peripheral hormones like catecholamines, and immune malfunction; however, until now, the diagnostic approaches are very limited. Virus reactivation and signs of inflammation are also common in severely overtrained athletes.

Carbohydrate metabolism is involved with insulin resistance and increased catabolic hormones like cortisol. Signs of disturbed carbohydrate metabolism are low leptin levels, insulin resistance and reduced maximum lactate. The stress hormones like cortisol or catecholamines are increased in acute situations, however, when fatiguing, the peripheral tissues decrease hormonal receptors which is counter-regulated by increased levels of hypothalamic release hormones, in severe cases the hypothalamic-peripheral axes are disturbed. Metabolic stress will reduce early sex hormone levels like estrogens and testosterone and the release hormones FSH and LH, peripheral thyroid hormones are down regulated as well as TSH.

There are parallels between the molecular mechanisms of ‘overtraining syndrome’ and systemic inflammatory reactions in trauma or sepsis. In healthy athletes, training induces a state of acute inflammation, which is rapidly counter-regulated by anti-inflammatory mechanisms. In the fatigued athlete, these mechanisms are disturbed and this leads to chronic inflammation and reduced immune function. Training induces so-called *“damage-associated molecular patterns”* (damps). These include molecules like free DNA, heat-shock-proteins or uric acid, which are released from the damaged muscle, oxidative stress and immunological signaling of so-called “*pattern recognition receptors”* (PRRs). These processes can be analyzed in blood samples by measuring inflammatory cytokines IL-1β, IL-8 and TNF-α, whereas a typical anti-inflammatory cytokine is represented by IL-10.

## Practical approaches

We now understand much more of the training processes. In general, this knowledge should lead to an improved performance/recovery balance in athletes and therefore less underperformance and injury, which is the primary goal of any diagnosis. The immunological hypothesis has gained more importance; however, practical measurements in the training process are very limited due to laboratory needs and costs.

Therefore, key symptoms of UUPS or OTS are underperformance and/or reduced trainability and disturbed regeneration following a period of heavy training load. Mood and sleep disturbances can be evaluated with questionnaires. Physiological signs are increased heart rate at rest and during a given workload.

Rest and recovery measures are the most important treatment in early cases of UUPS/OTS. When the problems are prolonged, a clinical workup including selected blood parameters should be performed.
